# A Novel Suspension Formulation Enhances Intestinal Absorption of Macromolecules *Via* Transient and Reversible Transport Mechanisms

**DOI:** 10.1007/s11095-014-1303-9

**Published:** 2014-02-21

**Authors:** Shmuel Tuvia, Dori Pelled, Karen Marom, Paul Salama, Maya Levin-Arama, Irina Karmeli, Gregory H. Idelson, Isaac Landau, Roni Mamluk

**Affiliations:** Chiasma, 10 Hartom St., POB 45182, Jerusalem, 91450 Israel

**Keywords:** absorption enhancer, intestinal permeability, oral drug delivery, sodium caprylate, tight junction

## Abstract

**Purpose:**

Medium chain fatty acid salts promote absorption by increasing paracellular permeability of the intestinal epithelium. Novel oily suspension (OS) formulation disperses a powder containing sodium caprylate and macromolecules such as octreotide or fluorescent dextran (FD). Formulation safety, macromolecule absorption and pharmacokinetic (PK)/pharmacodynamic (PD) were evaluated.

**Methods:**

Octreotide/OS toxicity was evaluated in monkeys following 9 months of daily oral enteric-coated capsule administration. The OS permeation effect was also assessed in rats, using FD/OS and octreotide/OS preparations. Octreotide/OS effects on circulating growth hormone (GH) levels were also measured.

**Results:**

Safety assessment of octreotide/OS in monkeys after 9 months showed minor drug-related findings, comparable to the injectable octreotide. Octreotide exposure levels were similar across the treatment periods. In rats, OS facilitated FD permeation up to 70 kDa in a reversible, spatial and dose-dependent manner, independent of the intestinal dosing site. Following OS administration, the staining pattern of the tight-junction protein, ZO-1, changed transiently, and a paracellular penetration marker, LC-biotin, permeated between adjacent epithelial cells. Enteral octreotide/OS absorption was dose-dependent and suppressed rat GH levels.

**Conclusions:**

Oral octreotide/OS dosing was shown to be safe in monkeys. OS enhances intestinal absorption of active octreotide, likely by transient alteration of the tight junction protein complex.

## INTRODUCTION

Passive intestinal absorption of hydrophilic macromolecules such as proteins or peptides (> 3 amino acids) (1) is limited due to low intestinal permeability and intraluminal enzymatic degradation (2,3). In contrast, lipophilic molecules are more likely to undergo passive partition into epithelial cell membranes, and thus be absorbed *via* a transcellular route (4). Columnar epithelial cells, joined at the apical surface by the tight junction complex, serve as an intestinal barrier for paracellular permeation of water-soluble macromolecules. The tight junction complex consists of transmembrane proteins, such as occludin and claudins, and several cytosolic proteins (5,6). Barrier functions are dynamically regulated by extracellular stimuli that are both physiologic (sugars, amino acids, and minerals) and pathophysiologic (pathogens and toxins) (7,8).

The tight junction permeation is enhanced by dietary components and/or other naturally occurring substances affirmed as Generally Recognized As Safe (GRAS) such as- glycerides, acylcarnitines, bile salts, and medium chain fatty acids [*See* review in (9)]. Sodium salts of medium chain fatty acids (MCFAS) were also suggested to be permeation enhancers. The most extensively studied MCFAS is sodium caprate, a salt of capric acid, which comprises 2-3% of the fatty acids in the milk fat fraction (10). To date, sodium caprate is mainly used as an excipient in a suppository formulation (Doktacillin™) for improving rectal ampicillin absorption. The permeation properties of another dietary MCFAS, sodium caprylate (8-carbon), were shown *in vitro* to be lower when compared to sodium caprate (11–14). In the present investigation, sodium caprylate and macromolecules *viz*., octreotide, a peptidic drug (15), or dextran, a polysaccharide, were formulated in an admixture with other excipients in oil to generate an oily suspension (OS).

This study explored the safety of the novel OS in a Cynomolgus monkey chronic toxicity study. Pharmacokinetic (PK)/pharmacodynamic (PD) effects of the OS on peptide or polysaccharide molecules and the OS effect on intestinal cells were also investigated in a rat model.

## MATERIALS AND METHODS

### Study Treatments

#### Formulation of Powder in Oily Suspension

**Fig. 1:**
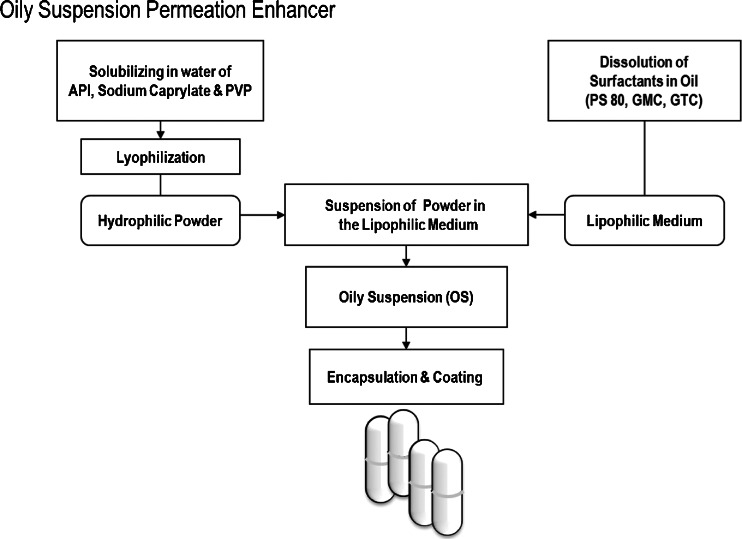
An oily suspention formulation of powder in lipophilic medium. Active pharmaceutical ingredient (API; *e.g*. octreotide acetate) was solubilized with sodium caprylate and polyvinyl pyrrolidone (PVP) in water. Next, the solution was milled, lyophilized and dispersed in lipophilic medium, comprised of polysorbate 80 (PS 80), glyceryl monocaprylate (GMC), and glyceryl tricaprylate (GTC). Drug/oil suspensions are then filled in capsules which are then enteric coated.

OS (TPE™; Chiasma, Jerusalem, Israel) was prepared using sodium caprylate as illustrated in Fig. 1 (16,17). Briefly, sodium caprylate (Merck, Darmstadt, Germany) and polyvinyl pyrrolidone (PVP; Kollidon 12, BASF, Burgbernheim, Germany) with or without octreotide acetate (Bachem, Torrence, CA) or fluorescein isothiocyanate (FITC)-labeled dextran (Sigma, St. Louis, MO) were solubilized in water, milled (particle size ranging 90 – 100 μm), lyophilized, and suspended in lipophilic medium consisting of polysorbate 80 (PS 80; Croda, UK), glyceryl monocaprylate (GMC; Abitec, Janesville, WI) and glyceryl tricaprylate (GTC; Abitec). The formulated OS was subsequently filled into hard-shell gelatin capsules (Capsugel, Bornem, Belgium) that were banded with prewarmed gelatin and 1% Twin 20 solution using a Dott Bonapace & Co. (Milan, Italy) semi-automatic banding machine, and dried at room temperature. The OS capsules were next enteric coated with a 20% aqueous suspension of Acryl-EZE® (# 93018509; Colorcon, Dartford, UK) using a perforated coating pan system (Compu-Lab, Thomas Engineering Inc., Hoffman Estate, IL) to reach a 90 mg capsule weight gain. The capsules were stored refrigerated (2 to 8°C) prior to their use.

**Fig. 2:**
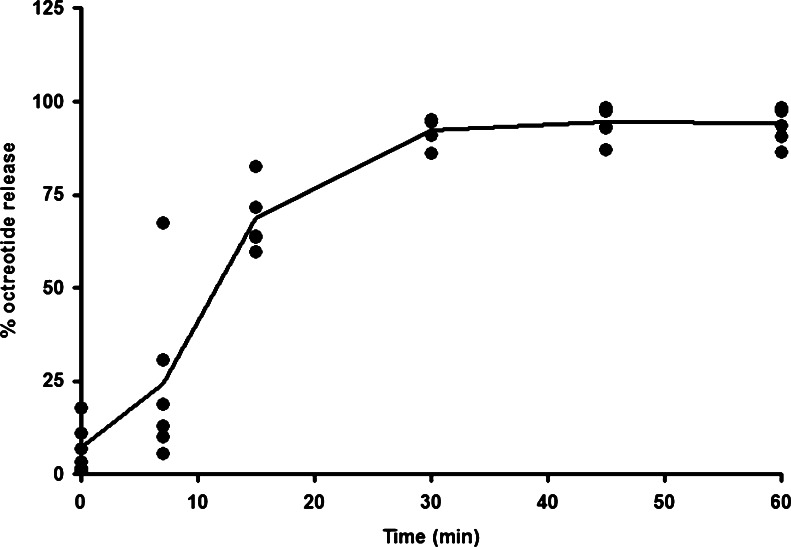
*In vitro* dissolution profile of enteric-coated gelatin capsules with 20 mg octreotide acetate and oily suspension. The *in vitro* release rate of octreotide from floating octreotide/OS enteric-coated capsules (*n* = 6) was determined using United States Pharmacopeia (USP) Dissolution testing apparatus II (two-stages, paddle method). Cumulative percentage of the octreotide release was calculated using an equation obtained from a standard curve.

The octreotide/OS capsules were tested for physical appearance, potency (assay), dissolution, microbiological and water content tests before their release and during the chronic toxicity study under refrigerated (2 to 8°C) and controlled room temperature (25°C and 60% relative humidity) conditions. The capsules appearance, white matt coated capsule, was maintained throughout the entire stability period. The octreotide content in the octreotide/OS capsules was analyzed by HPLC using Waters Alliance 2695 XE (Waters Corp., Milford, MA) equipped with Waters Photodiode Array Detector 2996 at λ = 210 nm. Briefly, the capsules were dissolved in 65 mM ammonium sulfate solution and analyzed by HPLC, using a C18 column (YMC Co., Ltd. Kyoto, Japan, 4.6 × 150 mm, 3 μm) with the following mobile phase: water (A), acetonitrile (B), 0.1% phosphoric acid and 20 mM sodium-1-decane sulfonate (C). The reported values were based on the signal area at the appropriate retention time. The octreotide content in the refrigerated octreotide/OS capsules confirmed with the drug specifications over the 9-month period. In addition, the drug release profiles from the enteric coated octreotide/OS capsules were carried out using a USP-II apparatus (Caleva 8ST dissolution apparatus, GB Caleva, Dorset, England) with a two-stage dissolution method according to USP <711> and Ph. Eur. 2.9.3. The tests were conducted in hexaplicates, in 900 ml dissolution medium maintained at 37 ± 0.5°C. A paddle speed of 50 rpm was employed. The tests consist of a 2-h acid stage dissolution in 0.1 N HCl (pH 1.2) with two sampling time points, and a 1-h buffer stage dissolution in pH 6.8 phosphate buffer with five sampling time points. The medium was changed by the analyst from the acid to the buffer after the first stage had been completed. The dissolution aliquots were withdrawn from the dissolution bath at the indicated time points and analyzed using the HPLC method for octreotide quantification. Samples were analyzed with a Phenomenex Luna C18 column (Phenomenex Co., Torrance, CA, 3.0 × 150 mm, 5 μm) at a flow rate of 0.6 mL/min and mobile phase containing acetonitrile-water-trifluoroacetic acid using Waters Alliance 2695 XE equipped with Waters Photodiode Array Detector 2996 at λ = 220 nm. Again, comparable dissolution profiles (Fig. 2) were obtained for the refrigerated octreotide/OS capsules during the 9-month period.

#### Animal Treatments

Enteric-coated capsules containing octreotide (20 mg)/OS or olive oil were administrated daily *per os* to fasted monkeys for 9 months, and swallowed with 10 mL water. In addition, a 1 mL ampule containing octreotide acetate at a concentration of 0.1 mg/mL (Sandostatin®, Novartis Pharmaceuticals, East Hanover, NJ) was administered by daily subcutaneous (SC) injection to another monkeys group. Oral administration of OS in rodent studies was limited due to the lack of properly enteric coated mini-gelatin capsules. Thus, the OS, containing fluorescent marker molecules (see below) or octreotide was enterally injected *via* implanted cannulas.

### Animals

#### Monkeys

Captive-bred, colony-raised, naive male and female Cynomolgus monkeys (*Macaca fasicularis*) were obtained from Covance Research Products, Inc. (Denver, PA) and acclimated for 9 days prior to treatment. In compliance with Good Laboratory Practices (GLPs), monkeys were housed individually in suspended stainless-steel cages. Room temperature was controlled to 18 – 29°C, humidity to 30% – 70% and cycled lighting (12 h of light daily). Prior to initiation of the study, the monkeys were in good health, and free of internal parasites and tuberculosis. Certified primate diet (Lab Diet # 5408 Primate Diet, PMI Nutrition International, Inc, Brentwood, MO) was provided twice daily (unless otherwise specified) on a feeding regimen of six to eight biscuits, supplemented by fruits (up to 5 per day). Water was provided *ad libitum*. Analysis for specific pathogenic microorganisms and contaminants showed no significant findings.

The study was carried out in an Association for the Assessment and Accreditation of Laboratory Animal Care International (AAALAC) accredited facility and in compliance with the Animal Welfare Act regulations (9 CFR 1). All aspects of this study were conducted in accordance with the Environmental Protection Agency and FDA based upon Good Laboratory Practice Regulations, 40 CFR 792.

#### Rats

Intestinal permeability experiments were performed *in vivo* using adult male albino Sprague–Dawley (SD) rats (300–400 g bodyweight), obtained from Harlan (Jerusalem, Israel) and acclimatized for 1 week in controlled temperature (22 ± 3°C) and 12-h light and dark cycles. Animals were housed individually, allowed access to rat chow (2018SC Harlan Teklad, Madison, WI) and tap water *ad libitum*. Animals were fasted for 16 to 18 h overnight before the start of the experiment, between 0700 and 0900 h. Study protocols and procedures were approved by the National Council for Animal Experimentation, Ministry of Health, Jerusalem, Israel.

### Chronic Toxicity Study in Cynomolgus Monkeys

#### Tissue Sampling and Preparation

At study initiation, monkeys weighing 3.34– 6.24 kg were randomly assigned (5/sex/group) to daily administration of single octreotide/OS capsule or control olive oil (negative control) by intragastric intubation or to SC injection of octreotide acetate (positive control) for 9 months. During the dosing period, animals were fasted for 8 to 10 h prior to dosing, and the first feeding was given 2 h after dosing. Monkeys were observed twice daily for mortality, morbidity, and clinical signs of adverse health effects and qualitative food consumption. Weekly examinations were performed on each animal before drug administration and before terminal sacrifice. Pre-dose body weights were recorded (week −1) and weekly thereafter. Blood samples (~5 ml) were collected for hematology and serum chemistry from the femoral vein before drug administration and from surviving animals prior to the terminal necropsy.

A complete ophthalmic examination was performed on each monkey by a Diplomat of the American College of Veterinary Ophthalmologist veterinarian before animal selection/group assignment (pretest) and again, prior to study termination.

Electrocardiograms (ECGs) were obtained twice pre-study and were analyzed by a veterinary cardiologist for the presence of significant abnormalities. ECGs were also obtained following the 1st dose and at the 3, 6, and 9 months of dosing. Standard ECGs (10 Lead) were recorded at 50 mm/s. Using Lead II (or another appropriate lead), the RR, PR, and QT intervals, and QRS duration were measured and heart rate was determined. The QT interval was calculated using Bazett’s Rule.

On the 270th day, the monkeys were fasted overnight, weighed on the following morning, and bled for required tests; then exsanguinated, necropsied, and examined for gross alterations. Animals were euthanized with intravenous ketamine sodium pentobarbital solution. Exsanguination was achieved by severing the femoral vessels.

#### Toxicokinetics

On Day 1 and after 3, 6 and 9 months, whole-blood samples (3.0 mL) were collected at routine intervals after completion of oral octreotide dosing. Plasma octreotide levels were determined by a validated liquid chromatographic spectrometric method (PPD Laboratories, Richmond, VA) as described elsewhere (18). The maximum plasma concentration (Cmax) and time to Cmax (Tmax) were derived from the data. The area under the curve (AUC) from zero to the last sample with a concentration ≥LOQ [AUC(0-t)] was calculated using the linear trapezoidal method. Analysis of plasma octreotide concentration *vs.* time was performed using SAS® for Windows® Version 9.3. The primary pharmacokinetic end point variables (Cmax and AUC_0-t_) were compared using analysis of variance (ANOVA) with treatment time (1, 180, 270-d) as a within-subject (repeated measures)-factor and sex as a between-subject factor.

### *In Vivo* Intestinal Permeability Assay

Intestinal paracellular permeability was determined by measuring the appearance, in the circulation, of a fluorescence marker, 4.4 kDa FITC-labeled dextran (FD4; Sigma). The assay of intestinal paracellular permeability was slightly modified from previously described methods (19–21). Briefly, implantation of jugular venous and intestinal catheters was carried out in SD rats under anesthesia, induced by intraperitoneal (*i.p.*) injection of ketamine hydrochloride (Kepro, Deventer, Holland) at 95 mg/kg bodyweight and xylazine hydrochloride (Sedaxylan; Eurovet Animal Health BV, Bladel, Holland) at 9.5 mg/kg bodyweight. Implantation of the catheters was carried out 5-7-days prior to the experiments. A thin heparinized polyurethane-based elastomer catheter (Micro-Renathane® MRE 025, 0.305 mm × 0.635 mm; Braintree Scientific Inc., Braintree, MA) was inserted *via* a 1-cm longitudinal skin incision, ventrally along the midline of the neck, into the right jugular vein. The proximal jejunum was localized immediately caudal to the ligamentum duodenocolicum, *via* a 2-cm ventral midline incision, and a silastic tubing (0.762 mm × 1.651 mm; Degania Silicone Co. Ltd, Degania Bet, Israel) tippet was inserted *via* enterotomy to the jejunum. Further experiments involved implanting an additional thin silastic tubing (0.500 mm × 1.000 mm; Degania Silicone) into the jejunum, adjacent to the 0.762 mm cannula. In a single series of experiments, a silastic catheter was inserted 2 cm distal to the pylorus (duodenum), 5 cm proximal to the cecum (ileum) or 1 cm distal to the cecum (colon). Both jugular and intestinal catheters were subcutaneously tunneled, exteriorized behind the animal shoulder blades and glued (histoacryl glue; B. Braun, Melsungen, Germany). Catheters were subsequently filled with heparinized saline and sealed. At the end of the surgery, all skin openings were closed with 0.5 silk sutures and stainless-steel wound clips.

For intestinal permeability experiments, 0.3 mL saline or OS containing 1.65 mg of FD4 was injected *via* the intestinal cannula into the lumen of conscious non-restrained rats. OS effect on permeability was also tested by administration of OS and fluorescent dextran using two jejunal cannulas, in which these marker molecule solutions in saline served as a “bystander” to the OS permeation activity. Additionally, in a single series of experiments, FITC-labeled dextrans of different molecular weights (average 10, 20, 40 and 70 kDa; Sigma) at an equivalent dose served as the permeability markers. Baseline blood samples (500 μL) were collected from the indwelling jugular catheter pre-dose, and at the indicated times. Blood was centrifuged at 4°C, 3,000×*g* for 10 min, and the plasma was separated for the analysis of FITC-dextran concentration. Plasma was diluted 1:1 with 5% sodium bicarbonate solution (Biological-Industries, Beit Haemek, Israel) in duplicate, and fluorescence intensity of the diluted plasma measured using a fluoro-spectrophotometer plate reader (Victor Wallac 1420 Multi-label; Perkin Elmer, Wellesley, MA) with an excitation wavelength of 485 nm and an emission wavelength of 520 nm. Plasma FITC-dextran levels are presented in arbitrary fluorescent units (AU).

### Immunohistochemistry

Experiments were conducted in anesthetized SD rats, using a ketamine-xylazine mixture administered *i.p.*, and animals were kept on warming pads (Bair Hugger®, Augustine Medical Inc, Eden Prairie, MN) to maintain body temperature at 38°C. The peritoneum was opened by midline incision and the proximal jejunum identified. After washing the intestines with 2 mL pre-warmed (37°C) saline solution, 0.3 mL saline solution or OS was injected 3 times (0, 10- and 20-min) into the animal lumen. At the indicated time-point, approximately 1-cm of the intestinal section was removed and washed with ice-cold phosphate-buffered saline (PBS).

Frozen sections of jejunal tissues were fixed with 4% paraformaldehyde in PBS for 2 h at 4°C, and then washed thrice with ice-cold PBS for 5 min. Next, jejunal sections were incubated with 30% (wt/wt) sucrose overnight in PBS at 4°C. Jejunal tissues were embedded in OCT cryostat freezing medium compound (Electron Microscopy Sciences, Hatfield, PA), snap-frozen in liquid nitrogen, cryostat sectioned (−80°C) at 14 μm, then mounted on Superfrost/Plus (Menzel Glaser GMBH, Braunschweig, Germany) microscope pre-coated slides.

Sections were incubated with monoclonal rabbit antibody against zonula occludens-1 (ZO-1; Zymed Laboratories Inc., San Francisco, CA) and against claudin-3 (Invitrogen Corporation, Carlsbad, CA) diluted at 1:100 in PBS/0.1% Tween-20 (PTW) for 72 h at 4°C. After washing thrice in blocking solution [Tween 20 (2%), heat inactivated goat serum (10%; Biological Industries, Kibbutz Beth Haemek, Israel) and PBS] for 5 min, sections were incubated with secondary Alexa Fluor 555-conjugated goat anti-rabbit IgG antibody (Invitrogen) at 1:200 for 1 h at 4°C. After washing thrice in blocking solution, sections were mounted on cover slides and images obtained using a laser-scanning confocal microscope (Zeiss LSM 510/Axiovert 100 M, Jena, Germany) and oil immersion objective (×40; NA 1.25).

Rat jejunal sections were also treated with sulfo-NHS-LC-biotin (Pierce, Rockford, IL) mixed with saline or OS and then washed, frozen and sectioned in a comparable procedure. Non-specific binding sites were blocked with blocking solution and 1 μg/mL non-labeled streptavidin (Zymed) was added for 1 h at room temperature. Sections were incubated for 30-min with 10 μg/mL of AlexaFluor-633 conjugated streptavidin (Invitrogen) in blocking solution. After washing thrice in blocking solution, sections were again incubated with the blocking solution and 1 μg/mL non-labeled streptavidin. Sections were then stained for actin by incubation with Alexa Fluor 555 Phalloidin (Invitrogen) diluted at 1:100 in the blocking solution for 30-min at 37°C. After washing thrice in blocking solution, sections were mounted on cover slides and images obtained using confocal microscopy.

### Immunoassays

Octreotide and growth hormone (GH) concentrations were assayed in rat plasma samples, following intra-jejunal dosing of 0.3 mL saline or octreotide/OS to cannulated rats. Octreotide acetate in saline was also injected subcutaneously to this animal model.

The octreotide immunoassay is based on a single-step, extraction-free, simultaneous assay procedure (Peninsula Laboratories, Division of Bachem, San Carlos, CA) designed to quantify octreotide concentrations. Briefly, octreotide standards (0.05–6.25 ng/mL) and rat plasma samples were diluted 1:10 with PBS, followed by addition of octreotide anti-rabbit antiserum antibody, biotinylated octreotide tracer, and assay buffer. Samples were washed and incubated with streptavidin–HRP. Optical density was read at a dual absorbance of 450 nm and 630 nm using an Enzyme-Linked Immunoassay (ELISA) reader (Multiskan EX Labsystems; Thermo Electron Corporation, Milford, MA) after addition of TMB solution and acidic stopping solution.

Plasma GH was measured by ELISA kit according to the manufacturer’s instructions (Diagnostic Systems Laboratories, Webster, TX).

### Data Analysis

Results were expressed as mean ± SE. The area under plasma concentration–time profile measured from time zero to the last measurable plasma concentration was calculated according to the linear trapezoidal rule. Relative enteral bioavailability (rBA) following intra-jejunal administration of the OS formulation to rats was calculated compared with SC injection as follows:

$$ rBA\%=\left({\mathrm{AUC}}_{\mathrm{jejunum}}/{\mathrm{AUC}}_{\mathrm{SC}}\right)\times \left({\mathrm{Dose}}_{\mathrm{SC}}/{\mathrm{Dose}}_{\mathrm{jejunum}}\right)\times 100 $$

Data were analyzed using ANOVA and a post hoc comparison between groups was conducted using the Student’s *t*-test (GraphPad, Inplot, San Diego, CA). A significance level of *P* < 0.05 was used for all statistical analyses.

## RESULTS

### Safety Evaluation of Oily Suspension

The effect of oral octreotide/OS administration on animal safety was evaluated in a 9-month toxicology study in Cynomolgus monkeys. No signs of toxicity were observed in the monkeys during and following daily administration of octreotide/OS for 9-months. Daily oral dosing of enteric coated OS-filled capsules with octreotide acetate for 9 months had no effect on bodyweight, electrocardiogram, ophthalmological, hematological (including coagulation), or clinical pathology (including Troponin I). Target organ toxicities based on macroscopic and/or histopathological analyses were not observed (*data not shown*). In the chronic study, increased incidence of sparse hair and red discoloured skin was observed in both sexes as well as a higher incidence of watery feces in the animals dosed with oral octreotide/OS and injectable octreotide acetate (positive control), compared to the negative control group (olive oil). Similar results were reported for the injectable octreotide formulation in the approved product label (Novartis, March 2012).

No treatment-related mortality was recorded in this toxicology study; however, three monkeys were euthanized or found dead (2 male negative control and 1 female positive control). These cases were attributed to complications in dosing procedures and are treatment-independent. More specifically, the olive oil capsule administration procedure in the negative control group, *i.e.* intragastric intubation, resulted in laryngeal obstruction with dosing capsule (*n* = 1) and pharyngeal swelling/thickening and hemorrhage (*n* = 1). In the positive control, accidental injury following octreotide injection resulted in fractured humerus and death. One female monkey from the negative control group was removed from the study due to a confirmed pregnancy. Thus, additional two animals were added later to this control group on Day 57.

No morbidity or mortality was documented following single or multiple OS dosing to a total of 284 conscious non-restrained cannulated SD rats.

### Oil Suspension Alters Intestinal Permeation *In Vivo*

**Fig. 3:**
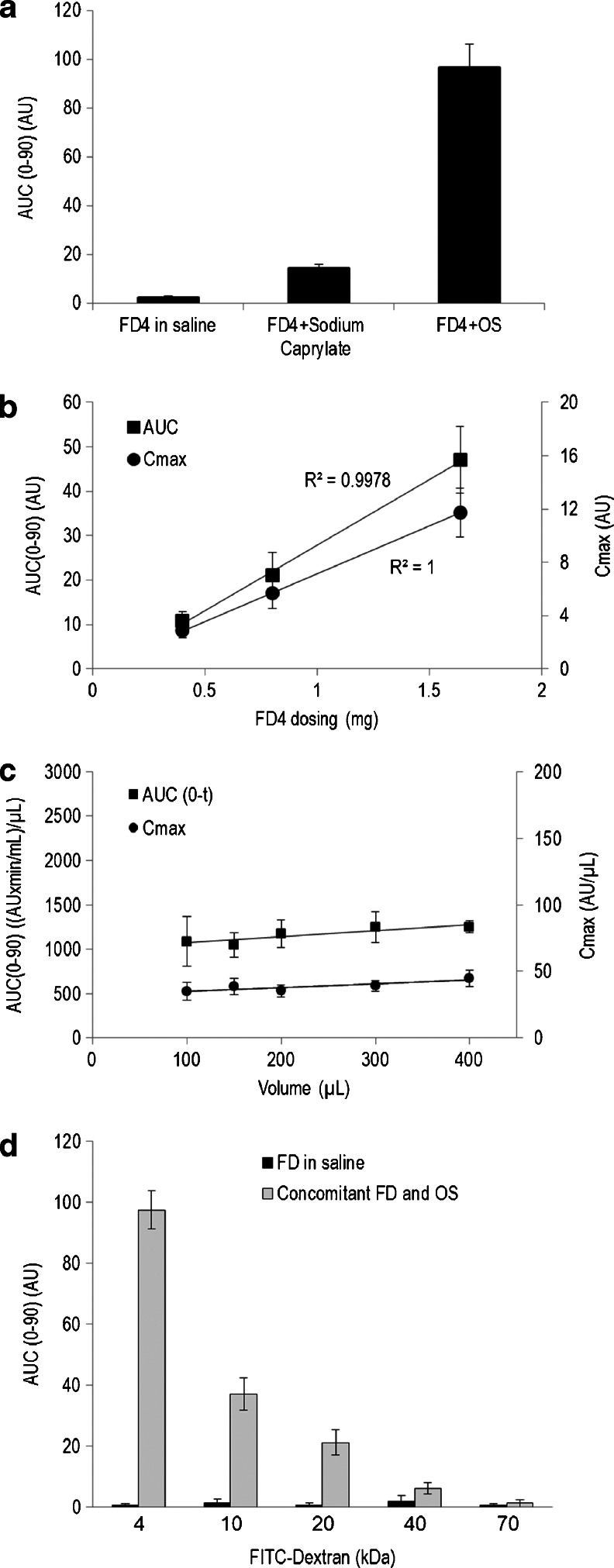
Permeability of FITC-labeled dextrans across the rat intestines. Plasma concentrations of fluorescein isothiocyanate (FITC)-labeled ~4.4 kDa dextran (FD4) were measured during 90-min in cannulated rats (**a**) following intra-jejunal administration of 1.65 mg FD4 in (*i*) saline, (*ii*) sodium caprylate in saline solution or (*iii*) in oily suspension (OS; Data represent Mean ± SE; *n* = 6–10). FD4 permeability was shown to significantly differ (*P* < 0.0001) between groups. (**b**) Rats were dosed with increasing concentrations of FD4 formulated in OS (0.3 mL). Data (Mean ± SE; *n* = 6) represent plasma FD4 area-under-the-plasma concentration time curve normalized to volume (AUC/volume; *squares*) and Cmax (*circles*). (**c**) A constant dose of FD4, formulated in increasing volume of OS (0.1, 0.15, 0.2, 0.3 and 0.4 mL) was administered to cannulated rats and plasma FD4 AUC (squares) and Cmax (*circles*) were plotted (Mean ± SE; *n* = 6-12). (**d**) Dextrans of increasing sizes FITC–dextran (FD) at 4.4, 10, 20, 40 and 70 kDa were solubilized in saline and concomitantly administered with OS (*gray bars*) or without OS (*black bars*) to cannulated rats and dextran levels in plasma measured. *Bars* represent means (± SE) AUC in arbitrary units (AU) over 90 min period of permeating experiments in 6–10 animals. Absorption of labeled dextrans, co-administered with OS, was significantly (*P* < 0.0001) higher than the absorption of FD dosed without OS.

Fluorescent marker (FITC-labeled dextran of 4.4 kDa; FD4) was solubilized in saline solution without or with sodium caprylate, or formulated with sodium caprylate in OS at a similar dose (1.65 mg). As expected, negligible increases in plasma FD4 levels were observed following administration of FD4 in saline to cannulated SD rats (Fig. 3a). However, significant increases in plasma FD4 levels were shown following dosing of a saline, FD4 and sodium caprylate solution to rats. An additional 5-fold increase in plasma fluorescent dextran levels was observed after administering formulated FD4 and sodium caprylate in OS. Addition of different FD4 concentrations into the OS (Fig. 3b) resulted in a dose-dependent effect on plasma levels. Dosing FD4/OS at a constant concentration and at increasing volumes (Fig. 3c) did not affect plasma FD4 levels. To examine effects of the molecular size on absorption, FITC-labeled dextrans of increasing sizes (4 – 70 kDa) were administered to rats concomitantly with OS. As demonstrated in Fig. 3d, elevation in plasma fluorescent dextran levels were shown to be inversely correlated with dextran size and insignificant permeating was detected at 70 kDa. Only low plasma fluorescent dextran levels were observed in all treated rats with the FITC-labeled dextran molecules solubilized in saline, irrespective of the molecular size of the administered marker.

**Fig. 4:**
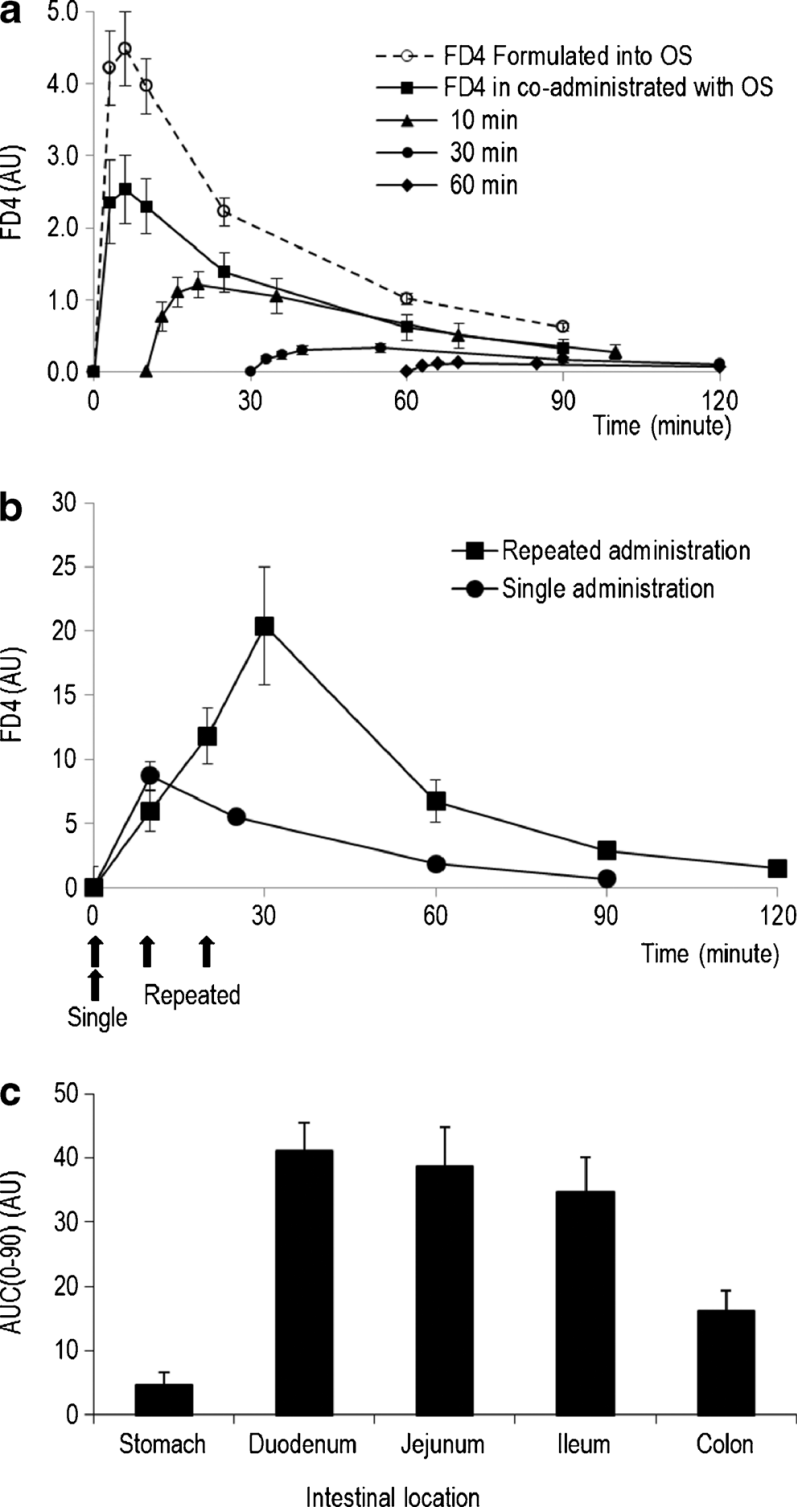
Duration of oily suspension-induced enteral absorption of FD4 in rats. The effect of oily suspension on intestinal permeability in cannulated rats was assessed by plasma 4.4 kDa FITC dextran (FD4) concentrations. (**a**) FD4 plasma concentration-time profiles upon enteral administration of FD4 formulated with oily suspension (OS; *dotted line*) or co-administered of FD4 in saline solution with OS (*arrow*) using adjacent jejunal catheters or FD4 dosing after 10-, 30- and 60-min (*arrowhead*) from the OS administration (Mean ± SE; *n* = 6–10). Plasma FD4 levels were shown to differ (*P* < 0.0001) between groups, using one-way ANOVA. (**b**) Intestinal permeability (plasma FD4) after a single or repeated (*arrows*) FD4 formulated in OS administration over 30-min experimental period was shown to be significantly different (*P* = 0.007; Mean ± SE; *n* = 6–10). (**c**) Apparent permeability across the rat digestive system (Mean AUC over 90-min ± SE; *n* = 6–10) of a single dose of FD4/OS *via* gavage to the stomach, or *via* catheter implanted at the duodenum, proximal jejunum, ileum and colon (*See* text for details). FD4 absorption was compared between groups using one-way ANOVA followed by post-hoc Tukey–Kramer pairwise comparison. Plasma FD4 levels differed (*P* < 0.0001) between groups, and post hoc analysis revealed that FD4 absorption in the stomach was lower (*P* < 0.05) compared with the duodenum, jejunum, ileum and colon groups.

To determine the duration of OS-induced tight junction permeation, change in plasma FD4 levels were compared in dosing of FD4 formulated in OS and co-administration of FD4 in saline and OS from two adjacent cannulas to SD rat jejunum (Fig. 4a). Both cases resulted in reversible FD4 absorption; however, formulated FD4/OS [AUC (0–90) 164 ± 14 AU] elicited a significantly higher elevation in plasma FD4 levels compared with levels observed with co-administration of FD4 and OS [AUC (0–90) 98 ± 20 AU; *P* = 0.021]. To examine the duration of permeation enhancement induced by a single dose of OS, administration of FD4 in saline 10, 30 and 60 min after the OS dosing (Fig. 4a) resulted in a significant and progressive reduction in FD4 plasma levels [10-min: AUC (0–90) 64 ± 15 AU, 30-min: AUC (0–90) 20 ± 3 AU, 60-min: AUC (0–90) 8 ± 1 AU; *P* < 0.001]. The limited FD4 absorption, upon dosing in saline 60-min after OS administration, may suggest that OS induces a transient intestinal permeation of about 1 h duration.

OS-induced permeation effect was tested for reproducibility over a short time period. Enhanced FD4 absorption was compared between a single and repeated (3-times) administration of FD4 formulated in OS. Figure 4b shows that plasma FD4 levels after administration of three consecutive FD4/OS doses, given every 10-min, was 2.6-fold higher (*P* = 0.007) then after a single dose [AUC (0–90) 90 ± 19 AU and AUC (0–90) 35 ± 7 AU, respectively]. The effect of OS was compared with administration of FD4/OS to the stomach (*via* gavage), the small and the large intestines of cannulated SD rats (Fig. 4c). Enhanced FD4 absorption after formulation in OS differed (*P* < 0.001) across the gastrointestinal tract. Post-hoc analysis suggested that FD4 absorption was significantly lower following gavage administration, whilst no differences in FD4 absorption were detected between the small and the large intestines.

**Fig. 5:**
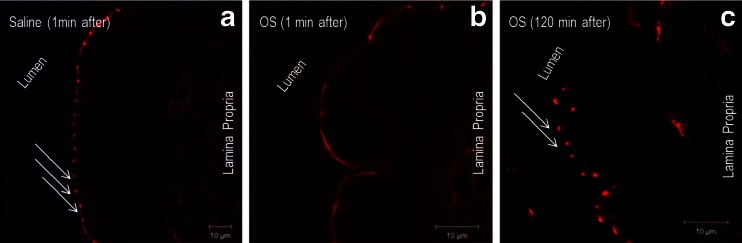
The oily suspension formulation induced transient reorganization of tight junction protein ZO-1. Frozen sections of SD rats proximal jejunum were labeled for ZO-1 (*red*) after 1 (**a**, **b**) and 120 (**c**) minutes from intra-jejunal administration of saline (**a**) or oily suspension (OS) (**b** and **c**). ZO-1 was stained at the apical junctions in jejunum from saline-treated rats, but presented with an altered staining pattern a minute after intra-jejunal OS dosing. At 120 min post OS administration, ZO-1 staining pattern was similar to that of saline treated rats. Data are representative of three independent experiments. Scale bar = 10 μm.

**Fig. 6:**
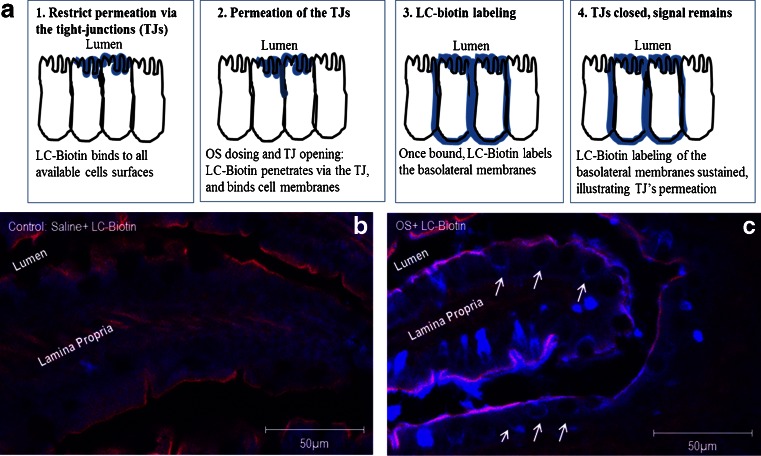
Paracellular permeability effect of the oily suspension formulation. To visualize the paracellular flux, sulfo-NHS-LC-biotin was added as a tracer to saline or oily suspension (OS). These preparations were then administered under anesthesia to the jejunum of SD rats (*n* = 3, each). Upon administration of LC-biotin tracer to the intestinal cells it is expected that the tracer staining will represent the intestinal permeation, inasmuch as that restricted permeation *via* the intestinal epithelium will present only staining on the mucosal layer whereas increased intestinal permeability will be translated into staining pattern that encompass also the basolateral membranes [see illustration in the *upper panel* (**a**)]. The *lower panel* images show the biotinylated tracer distribution (*blue*) by confocal microscopy in saline (**b**) and OS (**c**) treated rats. *Arrows* show the tracer permeated through the paracellular route in the OS but not the saline-treated rats. Lateral membranes are marked in *red* by actin and *purpl*e (overlay *blue* and *red*) indicates regions that the LC-biotin tracer penetrated between two adjacent epithelial cells. Scale bars represent 50 μm.

Effects of the OS formulation on the tight junction complex were next assessed using an immunohistochemical approach. The morphological changes of ZO-1, a tight junction protein, were evaluated by an immunofluorescent antibody labeling assay. As shown in Fig. 5a, ZO-1 was localized to the epithelial tight junctions in the jejunum of anesthetized control rats, which is appreciated as a series of bright red spots at the apical compartment of cell-cell junctions. In contrast, the ordered appearance of ZO-1 was altered in the jejunum of rats from the OS group. Specifically, 1 min following OS administration, the ZO-1 staining pattern was diffused through the luminal enterocyte membranes. The ZO-1 staining pattern of red spots was reestablished within 120 min from OS administration (Fig. 5c). Similar observations were shown for claudin-3 staining pattern prior to and following OS administration (*data not shown*). The effect of OS on the intestinal epithelium was further evaluated using sulfo-NHS-LC-biotin as a diffusion tracer according to the principle outlined in Fig. 6a. Images of control rat jejunum indicated that biotin diffusion tracer accumulated at the lamina propria (Fig. 6b). In contrast, in the OS group the tracer permeated through the intestinal barrier and was evident between the epithelial cells (Fig. 6c).

### Pharmacokinetics/Pharmacodynamics of Octreotide Formulated in Oily Suspension

**Fig. 7:**
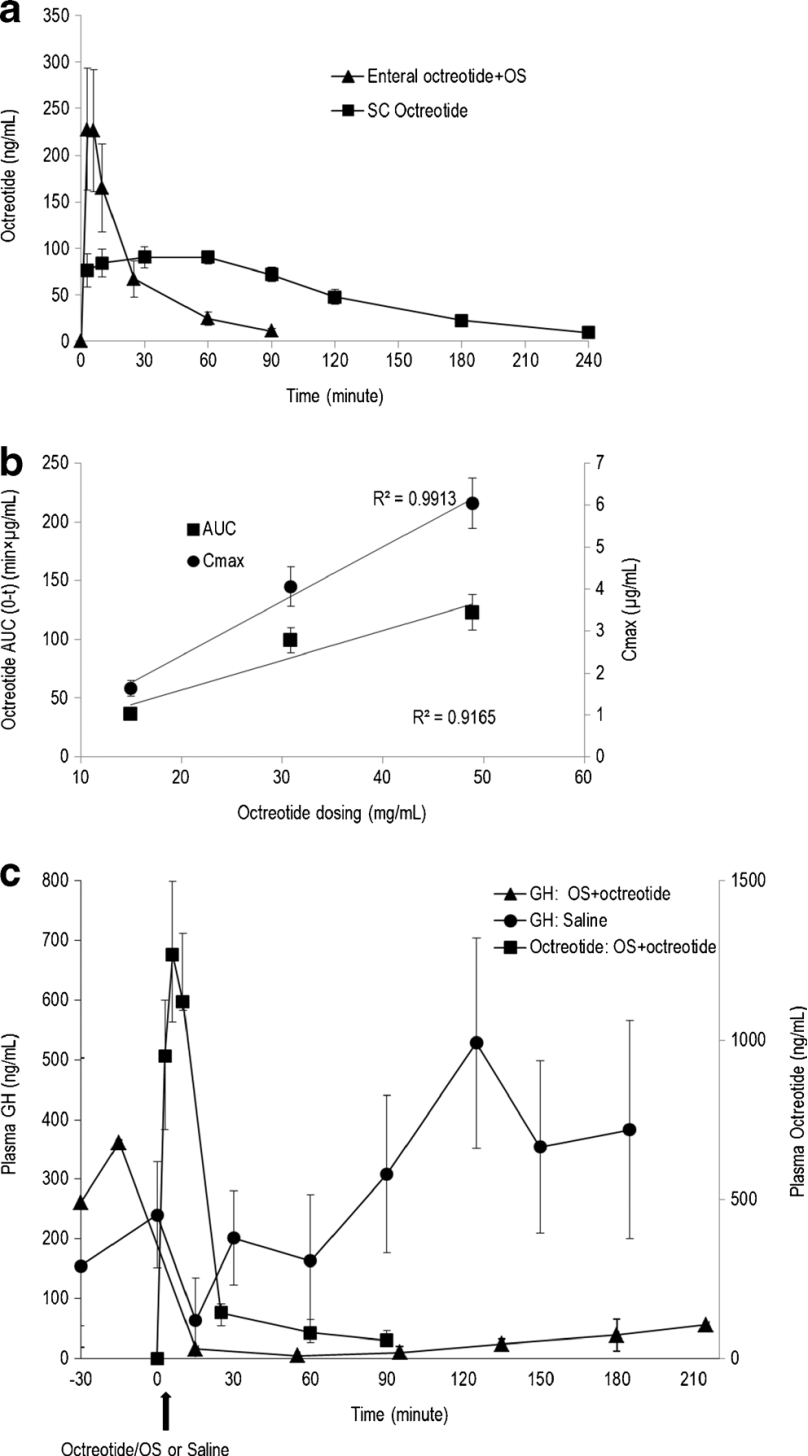
Enteral administration of octreotide formulated in oily suspension inhibits rat growth hormone levels. (**a**) Octreotide plasma concentration-time profiles after enteral administration of octreotide (3.2 mg/kg) formulated with oily suspension (OS) or after subcutaneous (SC) injection of octreotide acetate (0.172 mg/kg) in saline solution to cannulated rats (Mean ± SE; *n* = 5–12). (**b**) Maximum changes in the concentrations (Cmax; *circles*) and area-under-the-curve (AUC_0-t_; *squares*) of plasma octreotide after intra-jejunal administration of octreotide/OS formulation to male SD rats at increasing concentrations (Data are means ± SE; *n* = 9–1)]. (**c**) The effect of a single enteral administration (*arrow*) of saline (*circles*) or octreotide/OS (*triangles*) on growth hormones (GH) was evaluated in conscious SD rats. Blood was collected *via* jugular vein for GH analysis 30-min prior to dosing and up to 3.5 h post-dosing. In addition, octreotide concentrations (*squares*) were measured in plasma samples up to 90 min using ELISA assay (*See* text for details). Measured GH in the octreotide/OS-treated group were considerably lowered (*P* < 0.001) compared with the saline-dosed group and the pre-treatment levels (*P* < 0.001). Data are means ± SE (*n* = 10).

The toxicokinetics of circulating octreotide were studied following repeated oral administration of octreotide/OS in enteric-coated capsules to Cynomolgus monkeys in a 9-month toxicity and toxicokinetic study. Specifically, plasma samples for measurement of octreotide were collected prior to and up to 6 h following octreotide/OS or control olive oil capsules administration. Plasma octreotide concentrations were below the assay detection limits in the control animals (*data not shown*). Monkeys treated with octreotide/OS, however, exhibited significant plasma exposure to the investigational drug. Table I depicts the toxicokinetics of circulating octreotide following the 1st day and after 6- and 9-months of repeated daily oral octreotide/OS dosing. There was a consistent, though variable, exposure to octreotide during the 9 month study period, which was comparable in both male and female monkeys. The plasma octreotide levels after oral octreotide/OS capsule (20 mg octreotide) administration after the 1st day, 6- and 9-months (Table I) were comparable to the measured plasma octreotide levels after SC injection of octreotide acetate [0.1 mg; AUC (0-t) Day 1: Males 41.7 ± 8.6 h × ng/mL, Females 64.3 ± 5.3 h × ng/mL, Day 180: Males 46.2 ± 5.4 h × ng/mL, Females 60.8 ± 6.9 h × ng/mL, Day 270: Males 51.3 ± 11.3 h × ng/mL, Females 49.6 ± 3.0 h × ng/mL; average rBA of 2.3%]. The PK of octreotide/OS formulation was also evaluated in cannulated SD rats. Enteral dosing of octreotide acetate in saline solution resulted in negligible changes in rat plasma peptide concentrations (*data not shown*). Figure 7a shows the mean plasma concentration-time profile following a single intra-jejunal administration of octreotide/OS and after SC injection of octreotide acetate solution. After enteral administration of octreotide/OS, plasma octreotide levels rose rapidly, peaked after 6 min from dosing, and then fell below the limit of assay quantification after 90 min. Plasma octreotide levels after enteral octreotide/OS dosing [AUC (0–90) 14.6 ± 1.4 min × μg/mL] were comparable to levels observed after SC injection of octreotide acetate [AUC (0–90) 12.3 ± 0.4 min × μg/mL; average rBA of 7.4%]. Plasma octreotide concentrations after a single enteral administration of approximately 15, 30 and 50 mg/mL octreotide formulated in OS to conscious rats were dose proportional (Fig. 7b) for both the octreotide AUC *vs.* time as well as maximal plasma levels achieved.

**Table I:** Plasma Octreotide Levels of Cynomoglus Monkeys Orally Dosed for 9-months with 20 mg Octreotide Acetate and Oily Suspension in Enteric-coated Capsules

Sex	PK parameters	Treatment days		
		1	180	270
Male	Cmax (ng/mL)	22 ± 6	39 ± 35	30 ± 18
	AUC_0-t_ (h × ng/mL)	49 ± 13	84 ± 74	68 ± 40
Female	Cmax (ng/mL)	15 ± 4	13 ± 3	42 ± 14
	AUC_0-t_ (h × ng/mL)	34 ± 9	28 ± 7	92 ± 31
Total	Cmax (ng/mL)	19 ± 4	26 ± 13	36 ± 11
	AUC_0-t_ (h × ng/mL)	42 ± 8	56 ± 29	80 ± 25

Results expressed as arithmetic mean ± SE (*n* = 5 per gender)

The PK/PD relationship of octreotide/OS was investigated in cannulated rats administered intra-jejunal octreotide/OS or saline, and plasma octreotide and GH concentrations were determined (Fig. 7c). Octreotide PK parameters were in agreement with the aforementioned observations in rats. The time course of GH suppression in the octreotide/OS group was rapid and significant, as compared with pre-dose GH levels as well as with levels in the control saline group (Fig. 7c). GH suppression in the octreotide/OS group was sustained for >2 h after octreotide levels in plasma were low, *i.e.* below the detection limits.

## DISCUSSION

Enteral absorption of hydrophilic macromolecules under physiological conditions is restricted by the intestinal epithelium and especially its tight junctions. Thus, diverse permeation enhancers have been extensively studied as adjuvants for oral delivery of macromolecules such as peptides, which have inherently low oral bioavailability. In the present study, the safety of a novel OS formulation of the octapeptide octreotide was demonstrated in a monkey chronic toxicity study. OS permeation activity characterized in rats shows that OS transiently enhances permeability *via* paracellular transport in the small and large intestines. The OS was shown to affect distribution of the tight junction protein, ZO-1 in rat intestine. Enteral dosing of octreotide acetate formulated in OS to rats also resulted in systemic octreotide exposure and significantly attenuated plasma GH levels, demonstrating that octreotide retained its biological inhibitory activity.

The safety of octreotide acetate formulated with OS was demonstrated by the lack of clinical or laboratory evidence of adverse findings in monkeys after daily oral administration of enteric-coated capsules for nine months. Importantly, adverse macroscopic or microscopic findings were not detected in the gastrointestinal tract or liver in the toxicology study. These observations are also in agreement with the tolerance to oral octreotide acetate formulated in OS in a series of Phase 1 studies in healthy subjects (18).

The rat studies reported herein showed that OS permeation enhancement is accompanied by reorganization of tight junction proteins ZO-1 and claudin-3, and with diffusion of LC-biotin between enterocytes. Paracellular transport *via* the tight junction is physiologically regulated by intracellular signaling [See review in (22)]. Extracellular stimuli, including dietary components, drugs, and chemicals, alter paracellular transport (9). In the OS formulation, the key permeation enhancement excipient is sodium caprylate. The mechanism by which sodium caprylate affects the intestinal cells is not fully elucidated; however, the mechanism has been extensively studied for other members of MCFAS, such as sodium caprate. Lindmark and colleagues reported (23) that in Caco-2 cells, sodium caprate increased paracellular transport *via* contraction of actin filaments following phosphorylation of myosin light chains (MLC) by Ca2+/calmodulin-activated MLC kinase. Using this cell assay, others (24–26) have shown that tight junction dilatation following sodium caprate dosing was accompanied by altered localization of ZO-1, occluding, claudin-1 and F-actin. Kurasawa and colleagues (27) also reported that in human epidermal keratinocytes sodium caprate induced reversible redistribution of tight junction proteins, which was also associated with diffusion of LC-biotin *via* occluding-positive sites. Results shown here demonstrate for the first time effects of MCFAS *in vivo* on localization of tight junction proteins and the facilitation of paracellular penetration across the rat intestine.

To further characterize the permeation enhancement effect of OS, fluorescently labeled dextrans of known molecular sizes were formulated in OS and tested *in vivo* in rats. Dextrans were employed as marker molecules as they do not penetrate the intestinal wall nor are they metabolized (28,29). The FD4 permeation effect of OS was comparable between intestinal segments, but was limited following gavage administration. The latter finding, *i.e.* poor gastric FD4 absorption, may be associated with (*i*) effects of stomach acidity on the OS formulation, *i.e.* hydrolysis of the sodium caprylate to caprylic acid and so on for other excipients in the OS and/or (*ii*) limited OS permeation effect on gastric mucosa. These observations support the use of enteric-coated capsules for optimal intestinal permeation effect of the OS, *i.e*. to allow the OS to reach the small intestines without deterioration. Induced OS permeation effect was shown to be transient, non-refractory and reversible returning to baseline at about 1 h post dosing. In addition, the enhancing effect of OS was dose-proportional and molecular size-dependent. Absorption of fluorescent molecules decreased with increasing molecular weight from 4 to 40 kDa with only limited absorption occurring with 70 kDa dextran. Increased permeability was temporal, as demonstrated by decreased absorption of FD4 within 10 min after OS dosing. Intestinal permeability returned to basal levels approximately 1–1.5 h after OS dosing in rats. Taken together, the OS-induced intestinal permeation effect exhibits molecular size, spatial and temporal constraints.

The induced MCFAS permeation mechanism have raised safety concerns (10) for intestinal absorption of a gastrointestinal “bystander” pathogen. Current data in the literature would suggest the notion that increased intestinal permeability is insufficient to cause disease in an otherwise normal individual [*See* review in ref. (30)]. Moreover, transgenic mice with increased intestinal permeability or reduced intestinal barrier functions, exhibiting isolated intestinal epithelial tight junction barrier defects did not lead to spontaneous disease (31,32). These animal models suggest that the transient increase in epithelial permeability, such as resulting from tight junction regulation following OS administration, will not be pathogenic in an otherwise healthy host. This concept is further supported by the results shown here for the octreotide/OS safety in monkeys. In addition, the presented rat data suggests that the OS-induced permeation mechanism may prevent undesired absorption of pathogens residing in the intestines, which are typically ≥70 kDa.

Oral octreotide absorption from octreotide/OS in enteric-coated capsules was confirmed by toxicokinetic measurements in monkeys. Systemic exposure to octreotide was shown to be comparable between males and females and consistent, though variable, over the study period. These findings are in agreement with a previous report of administration of oral octreotide formulated with OS in enteric-coated capsules to healthy human subjects (18). Enteral octreotide acetate absorption enhanced in a concentration-dependent manner, when formulated with OS and administered to rats. In contrast, limited enteral octreotide absorption occurred in the absence of OS formulation, as previously shown by others (33–37). Effects of systemic octreotide exposure have been observed in cannulated rats. Rats dosed with octreotide/OS formula exhibited suppressed GH levels for >3 h post-dosing, whilst circulating GH concentrations in the control group were unaffected. These observations are consistent with the reported octreotide effect, *i.e*. binding to pituitary somatostatin receptors 2, 3, and 5 and markedly reducing GH secretion for several hours (38). Taken together, these results indicate that formulating octreotide with OS enables enteral absorption while maintaining potent somatostatin analog bioactivity in suppressing GH concentrations.

In conclusion, this study shows that daily oral administration of an octreotide/OS formulation for up to 9 months resulted in measurable plasma drug levels with minor treatment-related findings that were comparable to the octreotide injection. These results also demonstrate the feasibility of drug delivery using the OS in conjunction with hydrophilic macromolecules based upon permeation enhancer characteristics. A specific formulation of octreotide/OS is currently in a clinical study to evaluate the safety and efficacy of oral somatostatin analogue therapy in acromegaly patients.

## ABBREVIATIONS

AU: Arbitrary units
FD: Fluorescent dextran
GMC: Glyceryl monocaprylate
GTC: Glyceryl tricaprylate
MCFAS: Medium chain fatty acids
MLC: Myosin light chains
OS: Oily suspension
PS 80: Polysorbate 80
PVP: Polyvinyl pyrrolidone
rBA: Relative bioavailability
ZO-1: Zonula occludens-1
